# DEAD-box RNA helicase 21 negatively regulates cytosolic RNA-mediated innate immune signaling

**DOI:** 10.3389/fimmu.2022.956794

**Published:** 2022-08-10

**Authors:** Jia Li, Puxian Fang, Yanrong Zhou, Dang Wang, Liurong Fang, Shaobo Xiao

**Affiliations:** ^1^ State Key Laboratory of Agricultural Microbiology, College of Veterinary Medicine, Huazhong Agricultural University, Wuhan, China; ^2^ Key Laboratory of Preventive Veterinary Medicine in Hubei Province, The Cooperative Innovation Center for Sustainable Pig Production, Wuhan, China

**Keywords:** DEAD-box helicase 21 (DDX21), innate immunity, type I interferon (IFN-I), retinoic acid-inducible gene I (RIG-I), double-stranded RNA (dsRNA)

## Abstract

DEAD-box RNA helicase 21 (DDX21), also known as RHII/Gu, is an ATP-dependent RNA helicase. In addition to playing a vital role in regulating cellular RNA splicing, transcription, and translation, accumulated evidence has suggested that DDX21 is also involved in the regulation of innate immunity. However, whether DDX21 induces or antagonizes type I interferon (IFN-I) production has not been clear and most studies have been performed through ectopic overexpression or RNA interference-mediated knockdown. In this study, we generated DDX21 knockout cell lines and found that knockout of DDX21 enhanced Sendai virus (SeV)-induced IFN-β production and IFN-stimulated gene (ISG) expression, suggesting that DDX21 is a negative regulator of IFN-β. Mechanistically, DDX21 competes with retinoic acid-inducible gene I (RIG-I) for binding to double-stranded RNA (dsRNA), thereby attenuating RIG-I-mediated IFN-β production. We also identified that the 217–784 amino acid region of DDX21 is essential for binding dsRNA and associated with its ability to antagonize IFN production. Taken together, our results clearly demonstrated that DDX21 negatively regulates IFN-β production and functions to maintain immune homeostasis.

## Introduction

Innate immunity is the first line of host defense against viral infections and is triggered by pattern recognition receptors (PRRs). Several PRRs are mobilized to sense viral nucleic acids, ultimately leading to the induction of interferons (IFNs). Toll-like receptors (TLRs), retinoic acid-inducible gene I-like (RIG-I) receptors (RLRs), and cytoplasmic DNA receptors are the three main PRRs involved in the recognition of viral pathogen-associated molecular patterns (PAMPs). DEAD-box helicases (DDXs), which contain at least 12 conserved motifs, belong to helicase superfamily 2 and are a class of enzymes that are essential to all living organisms. DDXs are involved in various cellular processes (1, 2), including binding and unwinding nucleic acid strands (DNA, RNA, or RNA-DNA hybrids) (3). Additionally, growing evidence shows that DDXs are involved in innate immunity by sensing viral nucleic acids or regulating downstream signaling pathways (4, 5). RIG-I, also known as DDX58, is a member of the RLR as well as DDX family and responsible for the recognition of cytoplasmic double-stranded RNA (dsRNA); DDX41 recognizes intracellular DNA and bacterial cyclic dinucleotides (6); and DDX3 promotes downstream antiviral signaling by binding to the TANK-binding kinase 1 (TBK1) and inhibitor of kappa B kinase ϵ (IKKϵ) (7), all of which lead to IFN expression. Conversely, overproduction of IFN-I is detrimental and potentially lethal to the host, so preventing its harmful overproduction is essential to maintain the balance of innate and adaptive immunity. Several DEAD-box helicases have been identified that negatively regulate the production of IFN-I to maintain immune homeostasis. For example, DDX24 negatively regulates cytosolic RNA–mediated IFN-I signaling by competing with RIG-I for RNA (8), DDX19 inhibits IFN-I production by disrupting TBK1-IKKϵ- interferon regulatory factor 3 (IRF3) interactions (9), and DDX46 inhibits IFN production by entrapping m6A-demethylated antiviral transcripts, preventing their translation (10, 11).

DDX21, a well-known member of the DDX family, possesses all the characteristic motifs required for DEAD-box helicase function. DDX21 also contains an atypical FRGQR repeat at its C-terminus that contributes to its RNA binding and RNA folding activities (12), allowing it to directly bind rRNA and snoRNA and facilitate rRNA transcription, processing, and modification (13–16). Furthermore, DDX21 is responsible for the unwinding of RNA or RNA/DNA hybrid strands, such as dsRNA, R-loops, and G-quadruplex, depending on its ATPase activity, RNA helicase activity (17), and RNA folding enzyme activity (12, 18). Notably, DDX21 completes cellular processes through enzymatic, but it also exhibits non-enzymatic functions. The role of DDX21 in innate immunity has also been reported. A previous study showed that DDX21, together with DDX1 and DHX36, binds to the adaptor protein TRIF and senses dsRNA to activate the IFN signaling pathway, which is independent of RLRs. Briefly, DDX1 binds to dsRNA *via* the helicase A structural domain and then recruits DDX21 to mediate the interaction of DDX1 with DHX36. DDX21 and DHX36 bind to the TIR domain of TRIF *via* their PRK and HA2-DUF domains, respectively, and activate the IFN-I and NF-κB pathways. Knockdown of DDX21 negatively regulates poly (I: C), influenza A virus, or reovirus-induced IFN-I production (19). A recent study showed that DDX21 may balance viral/ligand-induced innate immune signaling by downregulating IFN-β expression. Vesicular stomatitis virus (VSV) infection and poly (I: C) treatment resulted in caspase-dependent cleavage of DDX21 at D126, facilitating its nuclear to cytoplasmic translocation. Cleaved DDX21 inhibits IFN-β production by preventing the assembly of the DDX1-DDX21-DHX36 complex, and knockdown of DDX21 inhibited IFN-β production, whereas overexpression of DDX21 did not affect IFN-β production. Interestingly, knockdown of DDX21 inhibits VSV replication and overexpression of DDX21 promotes VSV replication (20). In addition, overexpression of DDX21 has been reported to promote the activation of IFN-β promoters induced by dengue virus infection or background (21). Obviously, DDX21 antagonism or induction of IFN-I production remains controversial. Previous results on DDX21 regulation of the innate immune response were achieved by overexpression or small RNA interference assays. Moreover, the effect of DDX21 knockout on the innate immune response and whether DDX21 is involved in RLR-mediated innate immunity remains unclear.

In this study, a DDX21-knockout HEK-293T cell line was constructed by CRISPR/cas9 technology. DDX21 was shown to negatively regulate the production of IFN-β. Further analysis revealed that DDX21 inhibits dsRNA-mediated activation of Interferon regulatory factor 3 (IRF3) and NF-κB. Furthermore, we demonstrated that DDX21 is a dsRNA binding protein that antagonizes RIG-I recognition of viral dsRNA and attenuates dsRNA-mediated RIG-I signaling, leading to inhibition of IFN-β production.

## Material and methods

### Viruses and cells

Sendai Virus (SeV) was obtained from the Centre of Virus Resource and Information, Wuhan Institute of Virology, Chinese Academy of Sciences. VSV-GFP was gifted by Zhigao Bu at the Harbin Veterinary Research Institute of the Chinese Academy of Agricultural Sciences. HEK-293T cells were obtained from the China Center for Type Culture Collection (CCTCC) and cultured at 37°C in 5% CO_2_ in Dulbecco’s modified Eagle’s medium (DMEM/High glucose; HyClone, USA) supplemented with 10% heat-inactivated fetal bovine serum (FBS; PAN, USA) and 1% antibiotics (penicillin and streptomycin).

### Plasmid construction

The DNA fragment encoding DDX21 was amplified from cDNAs of HeLa cells and cloned into the pCAGGS vector with an N-terminal Flag or hemagglutinin (HA) tag to generate the expression plasmids pCAGGS-Flag-DDX21 and pCAGGS-HA-DDX21. The mutants of DDX21 (DDX21-K236E, DDX21-S375L, and DDX21-M4) were constructed by site-directed mutagenesis and named pCAGGS-Flag-DDX21-K236E, pCAGGS-Flag-DDX21-S375L and pCAGGS-Flag-DDX21-M4, respectively. A series of wild-type DDX21 truncation mutants were amplified by overlapping extension PCR using pCAGGS-Flag-DDX21 as a template and then cloned into the pCAGGS vector with an N-terminal Flag. IFN-β-Luc, NF-κB-Luc, and IRF3-Luc vectors have been described previously (22). The expression plasmids for Flag-tagged RIG-I, melanoma differentiation associated gene 5 (MDA5), Mitochondrial antiviral-signaling protein (MAVS/IPS1), TBK1, IKKϵ, and IRF3 have also been described previously (23).

### Dual-luciferase reporter assays

HEK-293T cells cultured in 24-well plates were co-transfected with 1 µg of the indicated expression plasmids or empty plasmids, 0.1 µg reporter plasmids (IFN-β/IRF3/NF-κB), and 0.02 µg pRL-TK reference plasmid (Promega, Madison, Wisconsin, USA) using jetPRIME (Polyplus, Illkirch, France) following the manufacturer’s instructions. At 24 h post-transfection (hpt), the cells were infected with SeV (10 hemagglutinating activity units/well) or transfected with poly (I: C) (*In vivo* Gen, Carlsbad, CA, USA) for 12 h. Firefly and Renilla luciferase activities from lysed cells were evaluated using the dual-luciferase reporter assay system following the instructions described by the manufacturer (Promega). Representative data from three independently conducted experiments were examined and data are expressed as the relative firefly luciferase activities with normalization to the Renilla luciferase activity.

### siRNA knockdown

The small interfering RNA (siRNA) targeting DDX21 (siDDX21) and negative control siRNA (NC) were designed and synthesized by Genepharma (Suzhou, China). The sequences used in our study are as follows: siDDX21, 5′-GCAUGAGGAAUGGGAUUGATATT-3′, and NC 5′-UUCUCCGAACGUGUCACGUTT-3′. HEK-293T cells with 60% confluency were transfected with 50 pmol of siRNA using jetPRIME (Polyplus) following the manufacturer’s instructions.

### RNA extraction and quantitative real-time PCR

Total cellular RNA was extracted using TRIzol reagent (Invitrogen) and 1 µg RNA of each sample was subsequently reverse transcribed to cDNA with the First Strand cDNA synthesis kit (Roche, Mannheim, Germany). The resulting cDNA was then used as the template for SYBR Green qPCR assay (Applied Biosystems, Foster City, CA, USA). The individual mRNA transcripts were assayed three times and relative mRNA expression levels were normalized to the expression of β-actin mRNA. All real-time PCR reactions were performed in an ABI 7500 real-time PCR system (Applied Biosystems). The qRT-PCR primers used in this study are shown in **Table S1**.

### Western blot analysis

Cells were lysed in lysis buffer (4% SDS, 3% dithiothreitol [DTT], 0.065 mM Tris-HCl [pH 6.8], 30% glycerin) supplemented with a protease inhibitor cocktail and a phosphatase inhibitor cocktail (Sigma, Shanghai, China). Equal amounts of proteins were separated by 10% SDS-PAGE and then transferred to a polyvinylidene difluoride (PVDF) membrane. The membrane was blocked with 5% nonfat milk in TBST with 0.1% polysorbate-20 followed by incubation with the indicated primary antibodies at 37°C for 3 h: rabbit anti-IRF3, p-IRF3, IKKϵ, p-IKKϵ, p65, p-p65, and RIG-I polyclonal antibodies (ABclonal, Wuhan, China); rabbit anti-STAT1, p-STAT1, STAT2, and p-STAT2 polyclonal antibodies (Cell Signaling Technology, Boston, USA); rabbit anti-DDX21 polyclonal antibodies (NOVUS, St. Louis, Missouri, USA); and mouse anti-β-actin, anti-Flag, and anti-HA antibodies (MBL, Beijing, China). After three washes in TBST, the membranes were incubated with horseradish peroxidase (HRP)-conjugated secondary antibodies (Beyotime, Shanghai, China) for 1 h at room temperature. Following washing, the membrane was visualized using enhanced chemiluminescence (ECL) reagents (BIO-RAD, USA). Expression of β-actin was used as a loading control.

### Co-immunoprecipitation assay

HEK-293T cells were co-transfected with expression plasmids encoding HA-DDX21 and Flag-RIG-I for 30 h. Cells were harvested and lysed on ice with lysis buffer (50 mM Tris-HCl, 150 mM NaCl, 1% NP-40, 10% glycerin, 0.1% SDS, and 2 mM Na2EDTA) for 30 min at 4°C. A portion of each supernatant from the lysed cells was used as whole-cell lysate (WCL). The remaining supernatants from the lysed cells were precipitated with mouse anti-HA monoclonal antibody (MBL) for 8 h at 4°C, followed by the addition of protein A+G agarose beads (Beyotime) for 4 h. The beads were collected by centrifugation at 3000 rpm for 5 min and washed three times with cell lysis buffer. The beads were resuspended in SDS-PAGE loading buffer and boiled at 95°C for 5 min. The samples and WCL samples were separated by SDS-PAGE and transferred to PVDF membranes, followed by western blotting analyses with the indicated antibodies.

### Poly (I: C) pulldown assay

HEK-293T cells cultured in a 60-mm plate were transfected with 3 µg of pCAGGS-Flag-RIG-I, pCAGGS-Flag-DDX21, or empty vector for 24 h. The cells were collected and lysed in 400 µl lysis buffer supplemented with a cocktail of protease inhibitors (Beyotime). The cellular lysates were mixed with the prepared poly (I: C)-coated agarose bead suspension and incubated at 4°C for 4 h. The beads were centrifuged and washed three times with 1 mL of lysis buffer, followed by western blot assay using mouse anti-Flag antibody as the primary antibody.

### Generation of DDX21 knockout cells

The CRISPR/Cas9 system was used to generate DDX21 knockout cells. DDX21-specific guide RNA (gRNA) was designed using the online CRISPR design tool (https://zlab.bio/). RNA oligonucleotides were annealed and cloned into the PX458 vector. The plasmid was transfected into HEK-293T cells for 36 h. Cells were digested with trypsin, and individual cells with green fluorescence were sorted by flow cytometry and inoculated into 96-well plates, ensuring one cell per well. Cell clones were sequenced to ensure that both alleles of the cell line contained frameshift mutations. In addition to PCR validation, western blot was also performed using the DDX21 antibody. The selected clones demonstrating unaltered DDX21 expression were used as wild-type (*DDX21^+/+^*) clones, while the clones deficient in DDX21 were used as *DDX21^-/-^* clones. The target sequences for guide RNA were as follows: DDX21 guide RNA 1: 5′-TTACAGTCCGGTTCAGGATG-3′, DDX21 guide RNA 2: 5′-AGTGAAGCTGCCAGTGAAGA-3′.

### Statistical analysis

Statistical analysis was performed using GraphPad Prism 8. Two-tailed unpaired t-tests were used to determine the significance of differences between groups. A *p-value* less than 0.05 was considered to be statistically significant.

## Results

### Knockout of DDX21 enhances cytoplasmic RNA-induced IFN-β production

**Figure 1:**
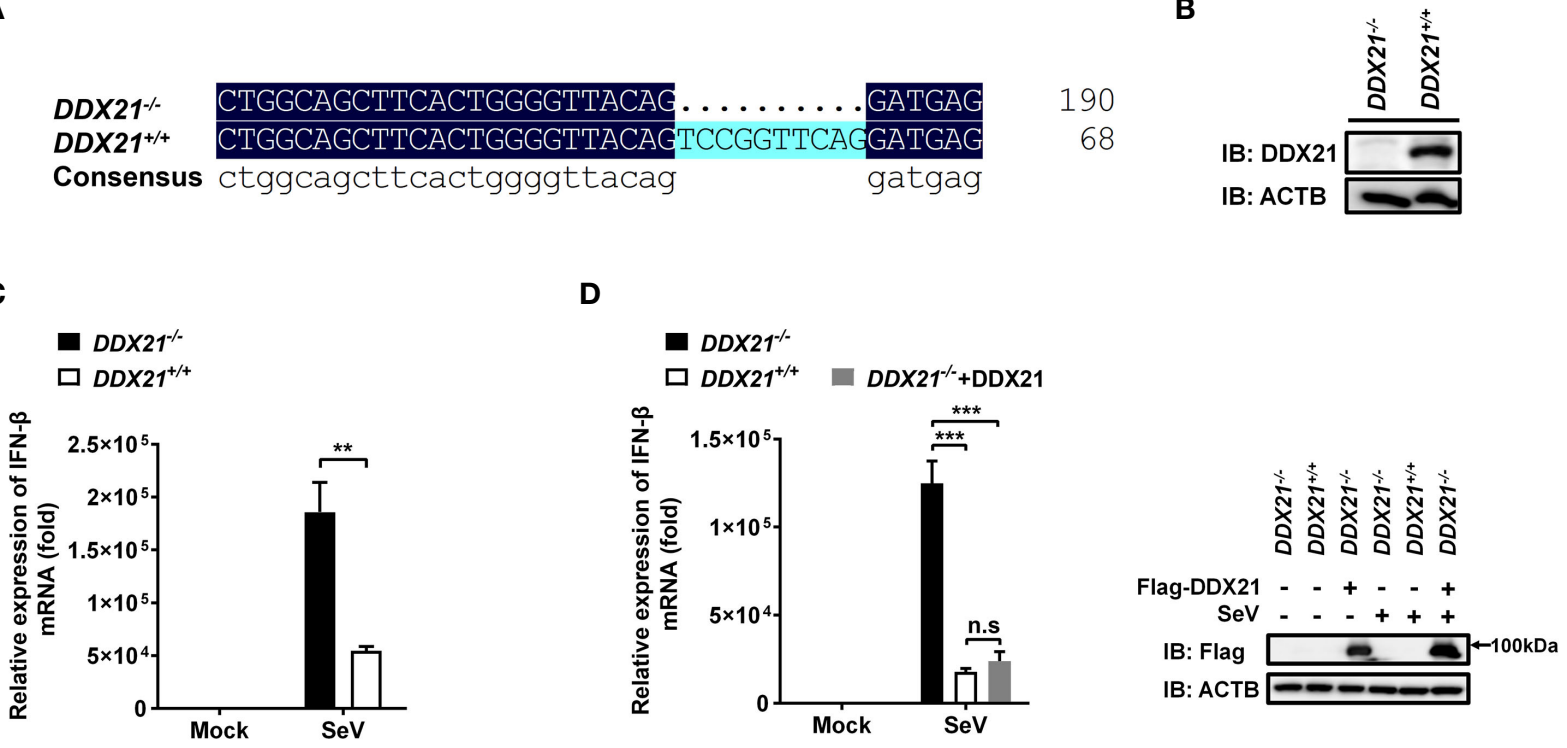
Knockout of DDX21 enhances cytoplasmic RNA–induced IFN-β production. **(A, B)** Genomic DNA and cellular lysates from *DDX21****^+/+^*** and *DDX21****^-/-^*** cells were subjected to sequencing PCR **(A)** and western blot assay with anti-DDX21 polyclonal antibody **(B)**. **(C)** *DDX21****^+/+^*** and *DDX21****^-/-^*** cells were seeded in 24-well plates, followed by infection with SeV (10 hemagglutination units/well). At 12 h post-infection (hpi), the cells were collected for qRT-PCR analysis for detection of IFN-β mRNA expression. **(D)** qRT-PCR analysis of IFN-β mRNA expression in *DDX21****^-/-^*** cells transfected with pCAGGS-Flag-DDX21 or empty vector followed by infection with SeV for 12 h Protein expression was evaluated by western blot assay (right blots). The experiment was repeated at least three times and the data shown are the means ± SD (n=3) of single representative experiments (**P < 0.01; ***P < 0.001). ns, not significant.

To ascertain the role of DDX21 in the IFN-I production pathway, we constructed DDX21 knockout HEK-293T cells using CRISPR/Cas9 technology with DDX21 guide RNA 1 editing. The knockout cell line HEK-293T *DDX21* ***^-/-^*** was confirmed at the DNA and protein levels (**Figures 1A, B**). Next, SeV-induced IFN-β production was analyzed by qRT-PCR in both *DDX21* ***^-/-^*** and *DDX2*1 ***^+/+^*** cells. As shown in **Figure 1C**, IFN-β mRNA expression was significantly upregulated in *DDX21* ***^-/-^*** cells compared with *DDX21* ***^+/+^*** cells. In addition, overexpression of DDX21 in *DDX21* ***^-/-^*** cells (a rescue experiment) also confirmed that DDX21 negatively regulates SeV-induced IFN-β production (**Figure 1D**). To exclude the off-target effect of CRISPR/Cas9, a DDX21 knockout cell line with DDX21 guide RNA 2 editing was used to assess IFN-β production. The qRT-PCR results showed that the deletion of DDX21 upregulated SeV-induced IFN-β mRNA expression, which was consistent with the results of **Figure 1C** (**Figures S1A, B**). These data suggest that DDX21 negatively regulates IFN-β production.

Given that DDX21 inhibits SeV-induced IFN-β production and that SeV as an RNA virus is a potent activator of the RLR pathway (24), we speculated that the inhibition of IFN-β is triggered by RNA. Poly (I: C) was used to assess the inhibition of IFN-β by DDX21 in the RNA-induced IFN signaling pathway. As shown in **Figure S2**, knockout of DDX21 significantly upregulated poly (I: C)-induced IFN-β mRNA expression. These data further demonstrate that DDX21 exerts a negative regulatory effect on cytoplasmic RNA signaling events in cells.

### DDX21 attenuates SeV-induced IRF3 and NF-κB activation

**Figure 2:**
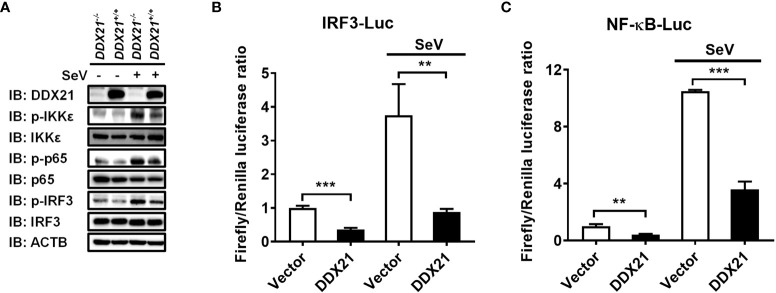
DDX21 attenuates SeV-induced IRF3 and NF-κB activation. **(A)** *DDX21****^+/+^*** and *DDX21****^-/-^*** cells cultured in 6-well plates were infected with SeV or left untreated for 12 h, followed by western blot analysis with primary antibodies against DDX21, total IKKϵ, total p65, total IRF3, phosphorylated IKKϵ, phosphorylated p65, phosphorylated IRF3, and β-actin. **(B, C)** HEK-293T cells were transfected with pCAGGS-Flag-DDX21 or empty vectors, together with IRF3-Luc **(B)** or NF-κB-Luc **(C)**, and pRL-TK receptor plasmids, followed by SeV stimulation for 12 h and dual-luciferase reporter assays. IRF3 -Luc **(B)** and NF-κB-Luc **(C)** contain four copies of the IRF3- or NF-κB-binding motif in front of a luciferase reporter gene, respectively. The experiment was repeated at least three times and the data shown are the means ± SD (n = 3) of single representative experiments (**P< 0.01; ***P < 0.001).

IRF3 and nuclear factor kappa B (NF-κB) are key transcription factors that mediate IFN production in the RLR pathway, and phosphorylated IRF3 and NF-κB subunit p65 (p-IRF3 and p-p65) are their activated forms (24). To analyze whether DDX21 affects the activation of IRF3 and NF-κB, *DDX21* ***^-/-^*** or *DDX21* ***^+/+^*** cells were stimulated by SeV, followed by western blot analysis. As shown in **Figure 2A**, DDX21 deficiency enhanced SeV-induced phosphorylation of IRF3 and p65. The same results were observed in DDX21 knockout cells edited by DDX21 guide RNA 2, excluding off-target effects (**Figure S1C**). HEK-293T cells were transfected with pCAGGS-Flag-DDX21 together with the luciferase reporter plasmids IRF3-Luc or NF-κB-Luc and pRL-TK, followed by dual-luciferase reporter assay. The results showed that SeV-induced IRF3 (**Figure 2B**) and NF-κB (**Figure 2C**) activation were compromised by overexpression of DDX21. These results strongly support a role for DDX21 as an IFN antagonist, blocking the activation of IRF3 and NF-κB in the SeV-induced RLR signaling pathway.

### DDX21 deficiency enhances anti-RNA viral responses

**Figure 3:**
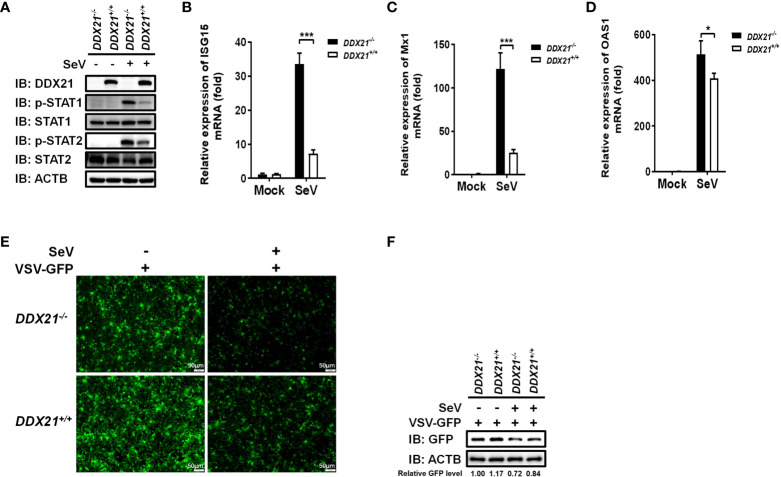
DDX21 deficiency enhances anti-RNA viral responses. **(A–D)** *DDX21****^+/+^*** and *DDX21****^-/-^*** cells cultured in 6-well plates were infected with SeV or left untreated. The cells were harvested at 12 hpi for western blot analysis with primary antibodies against DDX21, β-actin, total STAT1, total STAT2, phosphorylated STAT1, and phosphorylated STAT2 **(A)** or qRT-PCR for detecting the mRNA levels of *ISG15* **(B)**, *Mx1* **(C)**, and *OAS1* **(D)**. **(E, F)** *DDX21^+/+^* and *DDX21^-/-^* cells cultured in 24-well plates were infected with SeV or left untreated for 12 h The supernatant was harvested, treated by UV irradiation, and then overlaid onto fresh HEK-293T cells in 24-well plates. At 24 hpt, cells were infected with VSV-GFP. At 12 hpi, VSV-GFP replication was detected by fluorescence microscopy **(E)** or western blot with primary antibodies against GFP and β-actin **(F)**. Bar, 50 μm. The numbers below the images represent the rate of GFP to β-actin *via* ImageJ software analysis. The experiment was repeated at least three times and the data shown are the means ± SD (n=3) of single representative experiments (*P < 0.05; ***P < 0.001.

Several signal transduction pathways are involved in IFN production. Generated IFN-β binds to the interferon receptor and activates the JAK-STAT pathway, inducing the expression of interferon-stimulated genes (ISGs) to establish a cellular antiviral state (25, 26). To further investigate whether the deletion of DDX21 affects the expression of ISGs, we examined total and phosphorylated levels of key signaling molecules in the IFN signal transduction pathway. *DDX21* ***^-/-^*** and *DDX21* ***^+/+^*** cells were stimulated by SeV, followed by western blot analysis. As shown in **Figure 3A**, the phosphorylation levels of the above-mentioned signaling molecules were significantly up-regulated in SeV-infected cells compared with mock-infected cells. The expression levels of phosphorylated STAT1 (p-STAT1) and p-STAT2 were significantly up-regulated in *DDX21* ***^-/-^*** cells compared with *DDX21* ***^+/+^*** cells, but there was no difference in the total protein levels, confirming that knockout of DDX21 promotes SeV-induced RLR signaling. The effect of DDX21 knockout on SeV-induced mRNA expression of ISGs was further examined by qRT-PCR. As shown in **Figures 3B–D**, the mRNA levels of *ISG15*, *Mx1*, and *OAS1* were significantly upregulated in *DDX21* ***^-/-^*** cells compared with *DDX21* ***^+/+^*** cells. To further confirm the above results, we performed IFN bioassays using IFN-sensitive vesicular stomatitis virus expressing green fluorescent protein (VSV-GFP). Replication levels of VSV-GFP were inversely correlated with IFN-α/β levels secreted in *DDX21* ***^-/-^*** and *DDX21* ***^+/+^*** cells. IFA and western blot results showed that the supernatant of SeV-infected cells significantly inhibited the replication of VSV-GFP compared with the mock-infected cells, as demonstrated by the reduced green fluorescent signal and GFP expression. However, SeV-infected *DDX21* ***^-/-^*** cells supernatants inhibited VSV-GFP replication more strongly compared with *DDX21* ***^+/+^*** cells, as evidenced by the lower level of green fluorescent signal and GFP expression in the *DDX21* ***^-/-^*** cell group (**Figures 3E, F**). Taken together, these results demonstrate that DDX21 deficiency enhances anti-RNA viral responses.

### DDX21 fails to disrupt IFN-β promoter activation driven by RIG-I, MDA5, MAVS, TBK1, IKKϵ, or IRF3

**Figure 4:**
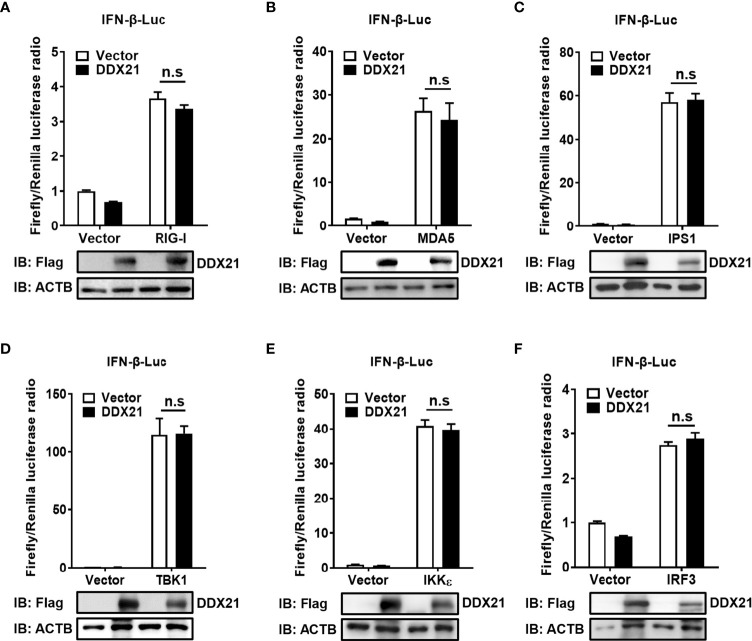
DDX21 fails to disrupt IFN-β promoter activation driven by RIG-I, MDA5, IPS1, TBK1, IKKϵ, or IRF3. **(A–F)** HEK-293T cells were co-transfected with IFN-β-Luc, pRL-TK, and pCAGGS-Flag-DDX21 along with constructs expressing RIG-I **(A)**, MDA5 **(B)**, IPS1 **(C)**, TBK1 **(D)**, IKKϵ **(E)**, and IRF3 **(F)**, respectively. Dual-luciferase reporter assay was performed 24 h after transfection. The relative firefly luciferase activity was relative to that of untreated empty vector control, with normalization of the Renilla reniformis luciferase activity. The expression of DDX21 protein was verified by western blotting with an anti-Flag antibody. β-actin served as a protein loading control. The results were repeated at least three times and the data shown are the means ± SD (n=3) of single representative experiments (n.s, no significant).

SeV is a strong inducer of the RLR-mediated IFN-β signaling pathway (24). Thus, the finding that DDX21 inhibits SeV-mediated activation of IRF3 and NF-κB suggests that DDX21 blocks the SeV-induced RLR-mediated IFN signaling pathway. To determine at which step of the RLR pathway DDX21 exerts its activity, we examined the effect of DDX21 on the production of IFN-β induced by a series of key signaling molecules in the RLR signaling pathway, including RIG-I (**Figure 4A**), MDA5 (**Figure 4B**), IPS1 (**Figure 4C**), TBK1 (**Figure 4D**), IKKϵ (**Figure 4E**), and IRF3 (**Figure 4F**). The results showed that in comparison with results in the corresponding empty vector-transfected cells, these key signaling molecules significantly induced IFN-β promoter activation, but the enhanced activation was not inhibited by DDX21 expression. These results suggest that DDX21 may act as a negative regulator of IFN-β either upstream of RIG-I or RIG-I level during SeV infection.

### DDX21 neither interacts with RIG-I nor affects its expression

**Figure 5:**
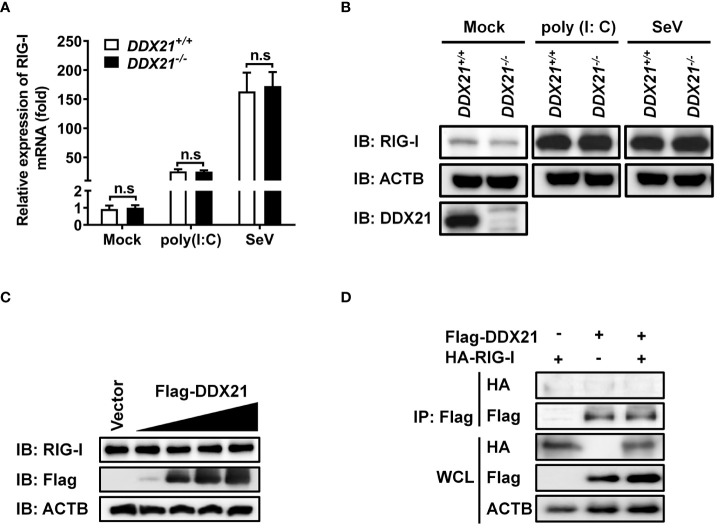
DDX21 neither interacts with RIG-I nor affects its expression. **(A, B)** *DDX21****^+/+^*** and *DDX21****^-/-^*** cells cultured in 6-well plates were stimulated with SeV or transfected with poly (I: C) for 12 h, followed by qRT-PCR and western blot assay for detecting the relative mRNA level **(A)** and protein expression **(B)** of RIG-I. **(C)** HEK-293T cells cultured in 6-well plates were transfected with increasing amounts (0.375, 0.75, 1.5, or 3 µg) of plasmids encoding Flag-DDX21. At 24 hpt, the cells were collected for western blot analysis with primary antibodies against RIG-I, Flag, and β-actin. **(D)** HEK-293T cells cultured in 60-mm dishes were co-transfected with expression plasmids encoding HA-DDX21 and Flag-RIG-I. At 24 hpt, the cells were lysed and subjected to immunoprecipitation analysis with an anti-Flag antibody. The whole-cell lysates (WCL) and immunoprecipitation (IP) complexes were analyzed by western blot using anti-Flag, anti-HA, or anti-β-actin antibodies. The experiment was repeated at least three times and the data shown are the means ± SD (n=3) of single representative experiments (n.s, not significant).

Both RIG-I (DDX58) and DDX21 belong to the DEAD-box helicase family and have a similar functional domain. To further determine whether DDX21 targets RIG-I level to affect its function, *DDX21* ***^-/-^*** or *DDX21* ***^+/+^*** cells in monolayer culture were treated with poly (I: C) or SeV for 12 h, followed by qRT-PCR and western blot assay for evaluation of the mRNA and protein levels of RIG-I. As shown in **Figure 5A** and **Figure 5B**, DDX21 knockout did not affect the mRNA and protein levels of RIG-I. Similar results were observed in cells transfected with various concentrations of DDX21 plasmid (**Figure 5C**). Moreover, co-immunoprecipitation results confirmed that DDX21 does not interact with RIG-I (**Figure 5D**). These results suggest that the inhibition of IFN-β production by DDX21 may occur at the RIG-I-dsRNA recognition step.

### DDX21 competes with RIG-I to bind dsRNA

**Figure 6:**
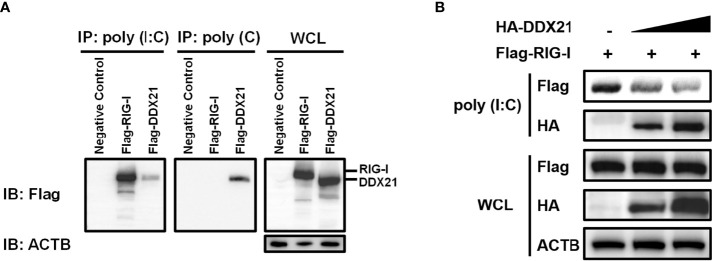
DDX21 competes with RIG-I for binding dsRNA. **(A) (A)** HEK-293T cells were transfected with expression plasmids encoding Flag-RIG-I, Flag-DDX21, or empty vector. Cells were lysed at 24 hpt and supernatant was incubated with poly (C)- or poly (I: C)-coated agarose beads for 4 h at 4°C. The beads were washed three times with lysis buffer by centrifugation, followed by western blot with an antibody against Flag. Poly (I: C)-coated agarose beads were prepared from poly (C)-coated beads (Sigma), which were incubated with 2 mg/ml of poly (I) (Sigma) for 1 h at 56°C. **(B)** HEK-293T cells were transfected separately with expression plasmids encoding Flag-RIG-I or HA-DDX21 for 24 h Cell lysates overexpressing DDX21 were incubated with an equal volume of cell lysates overexpressing RIG-I and then treated with poly (I: C)-coated agarose beads for 4 h at 4°C. The beads were washed three times with lysis buffer and then subjected to western blot analysis.

RIG-I activates the IFN signaling pathway by recognizing and binding dsRNA (27). DDX21 is an RNA binding protein that directly binds various RNAs (12, 13). Therefore, it is reasonable to speculate that DDX21 competes with RIG-I to bind dsRNA for inhibition of IFN-β production. To verify this hypothesis, we first examined the ability of DDX21 to directly associate with RNA species. An RNA pulldown assay was performed by poly (I: C)-coated agarose beads, which have been widely used to identify RNA-binding proteins such as MERS-CoV 4a and Ebola VP35 (28, 29). RIG-I served as a positive control because it has been proven to interact directly with poly (I: C) (30). As seen in **Figure 6A**, RIG-I bound to poly (I: C)-coated agarose beads but not bound to poly (C)-coated agarose beads, while DDX21 bound both poly (I: C)-coated agarose beads and poly (C)-coated agarose beads, indicating that DDX21 is both a dsRNA and single-strand RNA (ssRNA) binding protein.

To investigate whether DDX21 disrupts the binding of RIG-I to dsRNA, a competition assay was performed using poly (I: C) pulldown assays. HEK-293T cells were transfected with RIG-I plasmid or various concentrations of DDX21 expression plasmids for 24 h, and then the cells were collected for poly (I: C) pulldown analysis. The lysates from cells transfected with RIG-I expression plasmids were incubated with lysates from cells transfected with increasing concentrations of the DDX21 expression plasmid, followed by supplementation with prepared poly (I: C)-coated agarose beads for 4 h at 4°C. Bound RIG-I was then detected by western blotting. As shown in **Figure 6B**, the expression levels of RIG-I and DDX21 proteins were detected in WCL; however, significantly smaller amounts of RIG-I coimmunoprecipitated with poly (I: C)-coated agarose beads were detected with increasing concentrations of DDX21 protein. These results indicate that DDX21 at least partially functions to block the recognition or binding of dsRNA by RIG-I, leading to the antagonism of IFN-β production.

### The amino acids 217–784 of DDX21 are essential for binding dsRNA to inhibit IFN-β production

**Figure 7:**
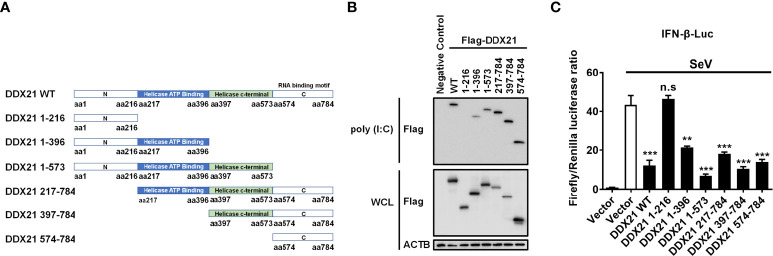
The aa 217–784 region of DDX21 is essential for binding dsRNA to inhibit IFN-β production. **(A)** Schematic representation of DDX21 WT and the truncation mutants. **(B, C)** HEK-293T cells were co-transfected with expression plasmids encoding Flag-tagged DDX21-WT or the truncation mutants. Cells were lysed at 24 hpt, and supernatants were examined in poly (I: C) pulldown assay **(B)** or dual-luciferase reporter assay **(C)**. The experiment was repeated at least three times and the data shown are the means ± SD (n=3) of single representative experiments (**P < 0.01; ***P < 0.001; n.s, not significant).

To determine the region of DDX21 responsible for binding dsRNA, a series of truncation mutants with N-terminal Flag tags were constructed on the basis of the reported functional domains of DDX21, which contains an N-terminal domain [amino acids (aa) 1 to 396], a Helicase ATP binding domain (aa 217 to 396), a Helicase c-terminal domain (aa 397 to 573), and a C-terminal domain (aa 574 to 784) that has been reported previously to be responsible for RNA binding (31, 32) (**Figure 7A**). HEK-293T cells were transfected with plasmids expressing Flag-tagged DDX21 or truncation mutants, followed by a poly (I: C) pulldown assay. As shown in **Figure 7B**, all truncation mutants were expressed at roughly equivalent levels in HEK-293T cells, and the pulldown assay demonstrated that the full-length DDX21, DDX21 1–396 (aa 1–396), DDX21 1–573 (aa 1–573), DDX21 217–784 (aa 217–784), DDX21 397–784 (aa 397–784), and DDX21 574–784 (aa 574–784) had the ability to bind dsRNA, but DDX21 1–216 (aa 1–216) lacked this ability. These results suggest that aa 217–784 of DDX21 are pivotal for dsRNA binding.

To further analyze whether the inhibition of IFN-β production by DDX21 is dependent on aa 217-784, HEK-293T cells were transfected with truncation mutants of DDX21, together with IFN-β-Luc and pRL-TK reporter plasmids, followed by stimulation with SeV, and subjected to dual-luciferase reporter analysis. As shown in **Figure 7C**, SeV stimulation notably induced the activation of the IFN-β promoter, but the increased activation was significantly lower in the presence of the full-length DDX21, DDX21 1–396, DDX21 1–573, DDX21 217–784, DDX21 397–784, and DDX21 574–784, suggesting that aa 217–784 of DDX21 are critical for suppressing SeV-induced activation of the IFN-β promoter. We conclude that DDX21 blocks IFN-β signaling by competing with RIG-I to bind dsRNA through the aa 217–784 structural domain.

### DDX21 antagonizes IFN-β independently of its ATPase, RNA helicase, and foldase activities

**Figure 8:**
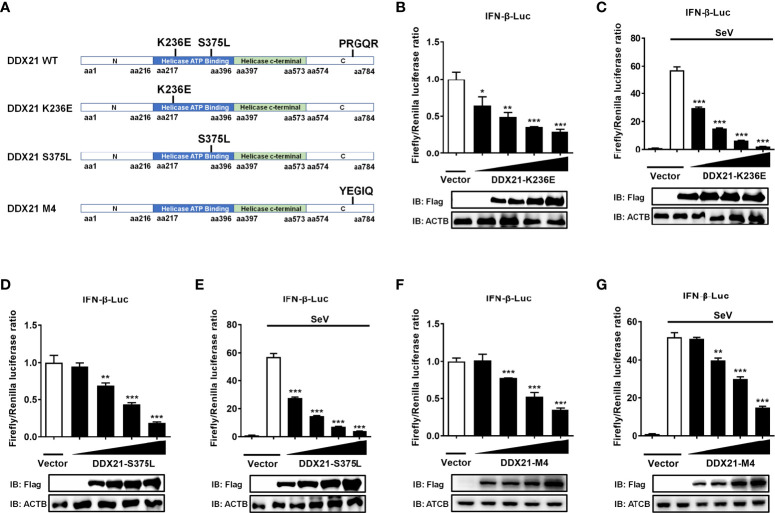
DDX21 antagonizes IFN-β independently of ATPase, RNA helicase, and foldase activities. **(A)** Schematic representation of DDX21 WT and mutants (K236E, S375L, M4). **(B–G)** HEK-293T cells cultured in 24-well plates were transfected with increasing amounts (0.1, 0.2, 0.4, or 0.8 g) of expression plasmids encoding Flag-DDX21-K236E, Flag-DDX21-S375L, or Flag-DDX21-M4, together with IFN-β-Luc and pRL-TK reporter plasmids for 24 h and then left untreated **(B, D, F)** or infected with SeV **(C, E, G)**. At 12 hpi, cells were lysed and the cell supernatant was collected for dual-luciferase reporter assay. DDX21 expression was examined by western blot with an anti-Flag antibody. β-actin served as a protein loading control. The results were repeated at least three times and the data shown are the means ± SD (n=3) of single representative experiments (*P < 0.05; **P < 0.01; ***P < 0.001).

DDX21 belongs to the DEAD-box RNA helicase family, and lysine 236 (Lys 236) and serine 375 (Ser 375) are highly conserved among DDX proteins and play key roles in its ATPase activity and RNA helicase activity, respectively. Mutating Lys 236 to glutamate (Glu) or Ser 375 to leucine (Leu) eliminates its ATPase activity or RNA helicase activity, respectively (33). In addition, DDX21 has an ATP-independent RNA foldase activity motif within the C-terminus, consisting of three repeating FRGQR sequences and one PRGQR sequence, and mutation of the PRGQR residues to YEGIQ has been previously shown to eliminate the foldase activity of DDX21 (17). To investigate whether DDX21 antagonization of IFN-β depends on its enzymatic activities, we constructed three enzyme activity-deficient mutants of DDX21: DDX21-K236E (only lacking ATPase activity), DDX21-S375L (only lacking RNA helicase activity), and DDX21-M4 (only lacking RNA foldase activity) (**Figure 8A**). HEK-293T cells were transfected with increasing doses of expression plasmids of these mutants, respectively. Dual-luciferase reporter assays showed that overexpression of the DDX21 mutants K236E, S375L, or M4 significantly inhibited IFN-β promoter activation in a dose-dependent manner with or without SeV stimulation, demonstrating that DDX21 inhibited IFN-β promoter activation independently of its ATPase, RNA helicase, and RNA foldase activities (**Figures 8B–G**).

## Discussion

DEAD-box RNA helicases are a class of ATP-dependent RNA helicases that are widely involved in various cellular RNA metabolism processes (34). They also play a crucial role in activating/inhibiting innate immune responses. Most DDXs are positive regulators of innate immunity (19, 35–38). However, increasing evidence suggests that DDXs also function as negative regulators involved in regulating excessive immune responses to maintain immune homeostasis (9–11, 20, 39). Here, we confirmed that DDX21 negatively regulates RLR-induced IFN-β production by knocking out DDX21 in HEK-293T cells using CRISPR/Cas9 technology. We found that DDX21 is a dsRNA/ssRNA binding protein that competes with RIG-I to bind dsRNA for inhibiting IFN-β production. The further study identified the key functional domain of DDX21 (aa 217–784) as essential for its ability to inhibit IFN-β production.

DDX21 has been implicated in viral RNA sensing as part of the innate immune response. However, previous reports on the effect of knockdown or overexpression of DDX21 on IFN production remain controversial. In one study, knockdown of DDX21 in dendritic cells inhibited poly (I: C), influenza A virus, and reovirus-induced activation of IFN-I and NF-κB pathways (19). Overexpression of DDX21 in HEK-293T cells promoted dengue virus-induced or background level activation of the IFN-β and ISRE promoters (21). Interestingly, a recent report showed that overexpression of DDX21 had no effect on IFN-β production but promoted VSV replication and that knockdown of DDX21 inhibited poly (I: C) and VSV-induced IFN-β production, as well as VSV replication (20). Notably, VSV has been reported to be an IFN-sensitive strain with a replication level that is inversely correlated with IFN expression (40, 41). In the present study, to exclude endogenous disruption, we knocked out DDX21 using CRISPR/Cas9 technology and investigated its effect on the IFN-β production. Our work confirmed that DDX21 negatively regulates IFN-β production; our findings using CRISPR/Cas9 technology provide more convincing results compared with previous reports using DDX21 knockdown or overexpression assays. In addition, we demonstrated that DDX21 knockout inhibited VSV-GFP replication, and IFN bioassay experiments confirmed that DDX21 knockout promoted IFN-β expression, which is consistent with previous reports. We speculate that the reason for the controversial findings may be due to the endogenous DDX21 expression and differences among various cell lines. Thus, further confirmation is needed by constructing DDX21 knockout cell lines in other cell types.

Previous studies reported that DDX21 induced the production of IFN-I mainly through the adaptor molecule TRIF (20, 42). However, how DDX21 affects the RLR pathway to regulate IFN-I production remains unclear. Viral RNA interacts with the helicase structural domain of RIG-I, inducing their conformational changes and allowing the CARD structural domain to interact with and activate MAVS, which drives the signaling cascade by activating the cytoplasmic kinases IKKϵ and TBK1, followed by the activation of NF-κB and IRF3. NF-κB and IRF3 act synergistically to induce IFN-I and other antiviral molecules (37, 43–45). Increasing evidence suggests that RIG-I is a target of immune regulation by various viral and host proteins. For example, murine hepatitis virus and SARS-CoV N proteins directly interact with the protein activator of protein kinase R (PACT), and the N-PACT interaction dissociates the association of PACT and RIG-I/MDA5, thereby inhibiting IFN-β production (46). Porcine reproductive and respiratory syndrome (PRRSV) N protein competes with TRIM25 for binding to RIG-I, inhibits the expression of TRIM25 and TRIM25-mediated ubiquitination of RIG-I, and antagonizes the production of IFN-β (47). Porcine deltacoronavirus (PDCoV) NS6 protein interacts with RIG-I/MDA5, which attenuates RIG-I/MDA5 binding to dsRNA, resulting in reduced RLR-mediated IFN-β production (48). DDX24 specifically binds to dsRNA to inhibit RIG-I-mediated signaling and negatively regulates IFN-I production. In this study, we confirmed that DDX21 can inhibit SeV-induced activation of IRF3 and NF-κB and negatively regulate SeV/poly (I: C)-mediated IFN-I signaling, Interestingly, DDX21 neither inhibited RIG-I-induced activation of the IFN-β promoter nor affected RIG-I expression but was able to bind dsRNA. A previous study demonstrated that the RIG-I CTD domain is responsible for selectively binding dsRNA (49) and that the expression of CTD inhibits RIG-I signaling and antagonizes SeV-induced IFN production (50). It is therefore plausible that DDX21 inhibits the SeV-induced RLR signaling pathway by competing with RIG-I for binding dsRNA. Competitive binding experiments with poly (I: C) pulldown also proved our speculation (**Figure 7B**).

The DDXs family of RNA helicases are a class of RNA-binding proteins. Previous reports have demonstrated that DDX21 can bind small nucleolar RNA (snoRNA), ribosomal (rRNA), and specific long non-coding RNA (lncRNA) (13, 14, 51). A recent report indicated that DDX21 interacts with G4 RNA and further confirmed that its C-terminal structural domain is necessary and sufficient for binding to G4 RNA (12). Our results indicate that DDX21 is a dsRNA- and ssRNA-binding protein with an RNA-binding region located at aa 217–784. Interestingly, gray values analysis of western blot shows that the C-terminus of DDX21 is the major dsRNA binding region, which is consistent with previous findings. In addition, previous studies have shown that RIG-I, MDA5, and Toll-like receptors 3 (TLR3) are the major PRRs that recognize dsRNA in the cytoplasm (52–57), while TLR7 and TLR8 are the major PRRs that recognize ssRNA in the cytoplasm (58, 59). Therefore, it is possible that DDX21 may function as a cytoplasmic RNA sensor, competing with other cytoplasmic PRRs to bind dsRNA/ssRNA independently of the DDX1-DDX21-DHX36 complex, thereby impairing signaling and negatively regulating immune responses. However, this possibility needs to be confirmed by further studies.

DDX21 has been shown to be a multifunctional enzyme with RNA helicase activity, ATPase activity, and RNA folding enzyme activity and is involved in almost all RNA-related processes (12, 16, 60). Moreover, we found that DDX21 interrupts RIG-I signaling by binding to dsRNA. Therefore, we explored the relationship between the enzymatic activity and dsRNA binding activity of DDX21 by constructing a series of function-deficient mutants of DDX21 to determine whether their effects on the IFN are related to its enzymatic activities. Unexpectedly, our results showed that the effects of DDX21 on inhibiting IFN-β production are independent of its ATPase, RNA unwinding, and RNA foldase activity.

In summary, we confirmed the inhibitory effect of DDX21 on dsRNA-induced IFN-β production using a DDX21 knockout HEK-293T cell line. Our results reveal a novel mechanism by which DDX21 negatively regulates IFN-β production by competing with RIG-I for binding to dsRNA through the aa 217–784 region. This study provides new insights into the function of the cytoplasmic dsRNA-binding protein DDX21 that negatively regulates the RLR pathway and maintains cellular homeostasis.

## Data availability statement

The original contributions presented in the study are included in the article/**Supplementary Material**. Further inquiries can be directed to the corresponding author.

## Author contributions

LF and SX conceived the study and designed the experiments. JL performed the experiments. JL performed the analysis. JL generated HEK-293T DDX21 KO cells and contributed to data interpretation. SX and JL wrote the manuscript. PF, YZ, and DW helped analyze the data. All authors contributed to the article and approved the submitted version.

## Funding

This study was supported by grants from the National Natural Science Foundation of China (no. 32130103, 31730095).

## Conflict of interest

The authors declare that the research was conducted in the absence of any commercial or financial relationships that could be construed as a potential conflict of interest.

## Publisher’s note

All claims expressed in this article are solely those of the authors and do not necessarily represent those of their affiliated organizations, or those of the publisher, the editors and the reviewers. Any product that may be evaluated in this article, or claim that may be made by its manufacturer, is not guaranteed or endorsed by the publisher.
