# Supplementary material for: DEAD-box RNA helicase 21 negatively regulates cytosolic RNA-mediated innate immune signaling
Source: Front Immunol. 2022 Aug 10;13:956794. doi: 10.3389/fimmu.2022.956794 (PMC9399600; doi:10.3389/fimmu.2022.956794)
Supplement: Supplementary file 1 [file DataSheet_1.docx]

Supplementary Material

# Supplementary Table

## Table S1

# The primer sequences for qRT-PCR

| **Gene name** | **Forward primer sequence (5′–3′)** | **Reverse primer sequence (5′–3′)** |
| --- | --- | --- |
| qDDX21 | AAACTCCGTAGTGACGCTGG | TCAACTTCAGAAGGCTCTGCTT |
| qIFN-β | TCTTTCCATGAGCTACAACTTGCT | GCAGTATTCAAGCCTCCCATTC |
| qGAPDH | TCATGACCACAGTCCATGC | GGATGACCTTGCCCACAGCC |
| qMx1 | ACCACAGAGGCTCTCAGCAT | CTCAGCTGGTCCTGGATCTC |
| qOAS1 | ACAGGCAGAAGAGGACTGGA | TAGAAGGCCAGGAGTCAGGA |
| qISG15 | TGGTGGACAAATGCGACGAA | CAGGCGCAGATTCATGAAC |
| qRIG-I | GCATGCATGGTGTTCCAGAT | TTCGTGCATGCTCACTGATAA |

# Supplementary Figures

## Figure S1


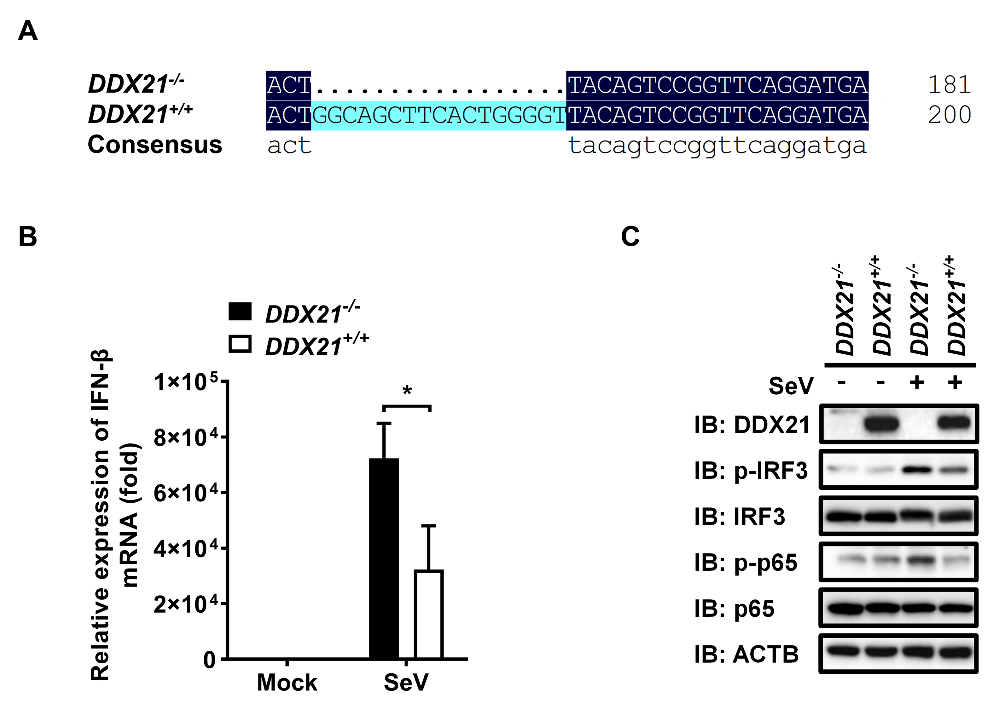


**Supplementary Figure 1.** A second DDX21 knockout cell line promotes SeV-mediated IFN-β production. **(A)** Genome editing was confirmed by sequencing PCR amplified fragments of the CRISPR/Cas9 target DDX21 genome from the cell lines. **(B)** *DDX21****^+/+^*** and *DDX21****^-/-^*** cells cultured in 24-well plates were infected with SeV. At 12 hpi, the cells were collected for use in qRT-PCR. **(C)** *DDX21****^+/+^*** and *DDX21****^-/-^*** cells cultured in 6-well plates were infected with SeV or left untreated. The cells were harvested at 12 hpi and analyzed by western blotting with primary antibodies against DDX21, β-actin, total IRF3, total p65, phosphorylated IRF3, or phosphorylated p65. The experiment was repeated at least three times and the data showed are the means ± SD (n=3) of single representative experiments (**P* < 0.05).

## Figure S2


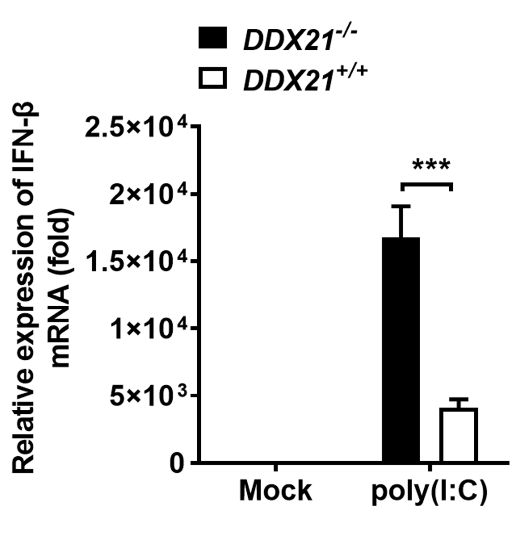


**Supplementary Figure 2.** Knockout of DDX21 increases poly (I: C)-induced IFN-β production. *DDX21****^+/+^*** *and DDX21****^-/-^*** cells cultured in 24-well plates were transfected with poly (I: C). At 12 hpt, the cells were collected for qRT-PCR. The experiment was repeated at least three times and the data showed are the means ± SD (n=3) of single representative experiments (****P* < 0.001).
